# Supplementary material for: Optimism and pessimism in optimised replay
Source: PLoS Comput Biol. 2022 Jan 12;18(1):e1009634. doi: 10.1371/journal.pcbi.1009634 (PMC8809607; doi:10.1371/journal.pcbi.1009634)
Supplement: Attachment — Submitted filename: Responses.pdf [file pcbi.1009634.s010.pdf]

Reviewer #1

> **Thank you for the opportunity to review this article. I have been interested in an article like this since seeing the Carey et. al (2019) paper as I thought it would be interesting to see how consistent / inconsistent the Mattar & Daw (2018) model would be with those results.**

Thank you very much for your overall assessment and helpful comments.

> **The authors conduct an impressive modeling analysis of pessimized replay in a (previously published) human behavioral dataset, arguing that pessimized replay is indeed optimal in the face of forgetfulness.**
>
> **My main critique has to do with some of the (potentially incorrect) assumptions in the article. I do think they can be addressed and thus hope to see this article published after clarification on these issues.**
>
> **There are two critiques here. Upon further deliberation, I think they are intimately related, but I will sketch them out separately and then try to offer a unified account.**
>
> **1. It's not clear to me that pessimized replay is inconsistent with the Mattar & Daw model as is (which the present authors discuss, albeit briefly, in lines 214-223). Consider the following case, in which there are 2 actions, L and R. The MB values are 1 and 10, respectively, whereas the MF values are 2 and 10. There is higher Gain (and thus EVB) when replaying the pessimistic action. Thus, replay can be used to devalue options, which was my interpretation of the Carey et. al (2019) finding. I consider this hypothesis to be simpler than the authors' forgetting hypothesis, and thus would like to see it ruled out first before considering more complex hypotheses.**

Indeed, the findings of Carey et al. 2019 are highly relevant to our work, as you kindly suggested. And we are not suggesting that our results are at all inconsistent with the Mattar & Daw mechanisms - they show what happens when Mattar & Daw occurs in the presence of forgetting in a combined model-free / model-based system.

Perhaps an illustration of your example in relation to our model would work best.

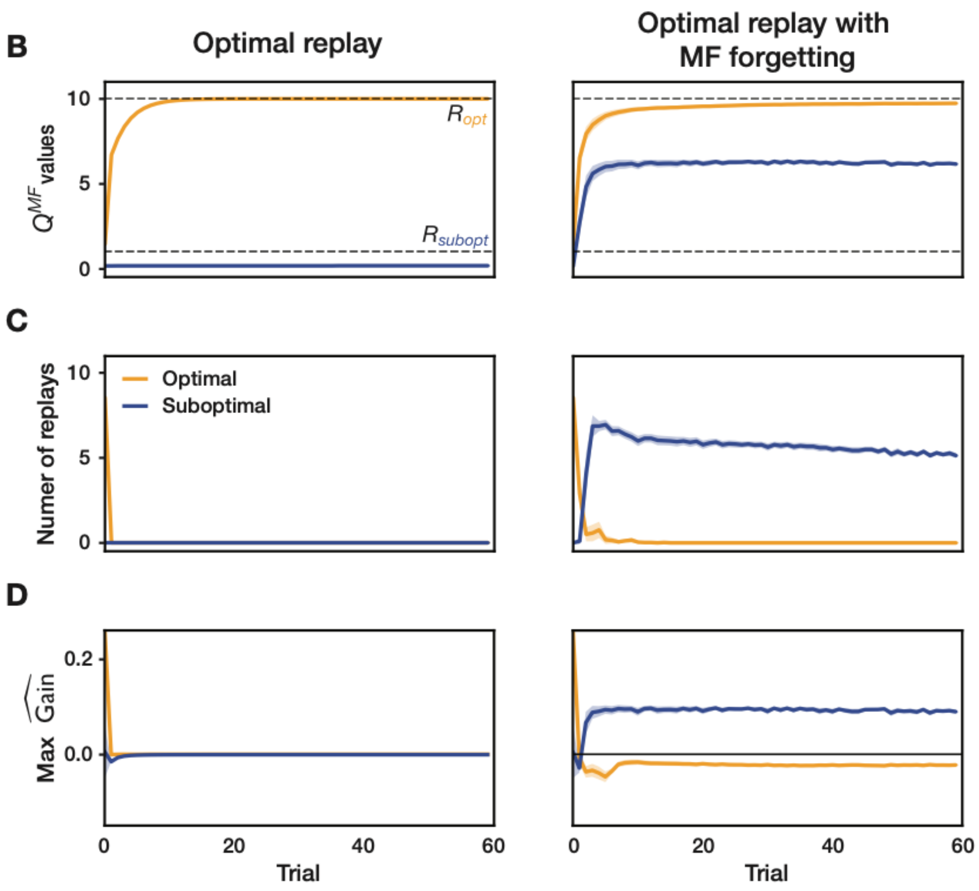

The above panels are taken from a new figure that we added (Figure 3 on page 7) to help motivate forgetting as suggested by our work, and moreover to help readers better understand the contribution of each individual model component.

The two agents (Q-learning with optimal replay and Q-learning with optimal replay and MF forgetting, with the former agent precisely implementing optimal replay as suggested by Mattar and Daw 2018) were simulated in a simple task involving only two actions – an optimal action which delivers a reward of 10 units (shown with orange lines) and a sub-optimal action with a reward of 1 unit (shown with blue lines).

We now explicitly state this connection to the optimal model of Mattar and Daw in lines 223-224:

'...such an unforgetful agent precisely implements optimized replay as suggested by Mattar and Daw 2018...'

Panel B shows the evolution of Q^{MF} values in both agents. Note how the Q^{MF} value estimates of the agent without MF forgetting quickly converge and remain stable, thus allowing the agent to act optimally. The agent with MF forgetting, on the other hand, experiences a situation in which continual learning (for instance, by means of optimal replay) is required to maintain good performance (since both Q^{MF} values decay towards the average reward experienced by the agent, and therefore replay in this case helps the agent distinguish which action is optimal and which is sub-optimal, on the level of MF values).

In panel C, we show what such MF forgetting implies for the agent's replay choices. The unforgetful agent does not engage in replay apart from in the very first few trials. This is because the agent's gain estimates (panel D; left) are virtually zero, since it is already apparent to the agent which action is optimal and which is sub-optimal. Note that, by contrast, the forgetful agent enjoys extensive replay of the sub-optimal action, since it estimates there to be substantial gain in devaluing the Q^{MF} value for that action in order to increase the estimated cumulative future reward.

Thus, you are indeed quite right about the positive gain for (and hence the incentive to replay) the sub-optimal action in the imagined simulation you suggested; however, MF forgetting is necessary for the Q^{MF} values for sub-optimal actions to be overestimated in the first place – otherwise the agent does not engage in persistent replay throughout acting in the task.

We added the above line of reasoning to our manuscript (lines 203-216), in addition to the figure from which those panels were taken, to help the readers understand the contributions of replay and forgetting to our model's behaviour before considering them in greater detail.

2. Second, I'm not sure I understand the authors idea of MF forgetting. From what I do understand, the problem the authors are identifying is the following. Since TD learning operates under the assumption that the optimal policy will be chosen after the first step, once the optimal policy is reached via training, the value (and thus the probability, assuming a softmax policy) of suboptimal actions will be inflated because the agent will make good decisions afterwards.

The authors say "Insofar as the agent improves over the course of the task, the average reward it obtains increases with each trial. MF values for sub-optimal actions, therefore, tend to rise towards this average experienced reward; MF Q-values for optimal actions, on the other hand, become devalued, as the agent is prone occasionally to choose sub-optimal actions due to its non-deterministic policy. In other words, because of MF forgetting, the agent forgets how good the optimal actions are and how bad the sub-optimal actions are."

Is the claim that this optimistic estimate of MF values is considered 'MF Forgetting'? This seems to be a different definition of 'forgetting' than what is commonly thought.

OR

Are the authors claiming that changing the temperature in order to inject more indeterminism into the policy is forgetting? This, I think, would be a more palatable interpretation of forgetting, but the rationale for this is lost on me, especially given that it runs counter to the concept of annealing.

My attempt at reconciliation in order to understand the authors' intuition (but this is surely to be incorrect, so please correct me) is that the authors have identified that the value of suboptimal actions becomes inflated due to the mathematical formalism of TD learning and thus replay may function as a mechanism to devalue these actions to prevent further use (and thus we see pessimistic replay). If this is indeed what is happening, I worry about using term 'forgetting' so prominently as I believe it has very different connotations than something like 'devaluation'. I understand I may

be harping on terminology here, so I do apologize; I do think it's an important distinction, though.

If I am incorrect in understanding the authors' intuition, please correct my mistakes.

The mathematical formalism of Q-learning does indeed lead to the overestimation of Q^{MF} *values due to the max operator, particularly in the case of (noisy) function approximators, as was identified by Thrun and Schwartz 1993. However, our Q-learning rules (equations 7, 8) did not involve the max operator, since all* Q^{MF} *values were updated towards the received reward, as opposed to the maximum over the values of the succeeding state (and we did not employ function approximation).*

Thus, MF forgetting in our work is not an inherent peculiarity of the Q-learning rule per se. We model MF forgetting as the decay of Q^{MF} *values towards the average reward experienced by the agent,* $\overline{r}$*. This follows the simple structure (introduced in Fig 2B):*

$$Q^{MF} \leftarrow (1 - \phi^{MF})Q^{MF} + \phi^{MF}\overline{r}$$

Forgetting in a form like this is quite common in the literature. All Q^{MF} *values thus relax towards* $\overline{r}$*, at a rate which is controlled by the MF forgetting parameter,* ϕ^{MF}*. The effect of MF forgetting, therefore, is not only limited to sub-optimal actions.*

Thus, consider again the above example of two actions that you suggested. Here, the true reward for the optimal action is 10. If the agent manages to persist in executing that action, albeit with occasional lapses to the sub-optimal action (due to the softmax stochasticity) – the average reward the agent experiences will be lower than 10 (i.e., the true value for the optimal action) – and the Q^{MF} *value for the optimal action will therefore tend to be devalued (as a result of our MF forgetting equation) towards that average experienced reward. In fact, this effect is visible from panel B (right) in the above figure – notice that the* Q^{MF} *value estimate for the optimal action in the forgetful agent is slightly lower than its true value (indicated with a dashed grey line). We also show this effect in Fig 4B:*

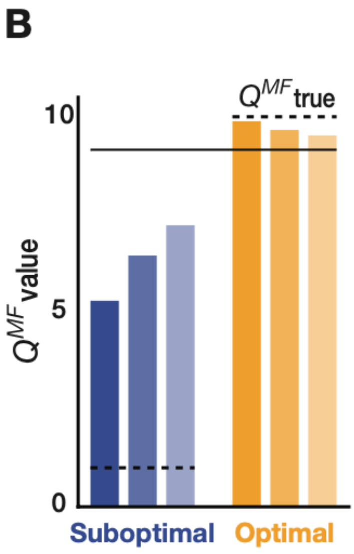

where, in addition, we show cases with increasing strengths of MF forgetting (the more transparent bars).

However, since the agent additionally learns on-line about the value of the optimal action, the effect of MF forgetting for this action is rather modest relative to that for sub-optimal actions, which are not visited as often. Thus, the values of sub-optimal actions are forgotten to a much more substantial degree (and so rise towards that same average experienced reward).

*Thus, to answer your question, '**Is the claim that this optimistic estimate of MF values is considered 'MF Forgetting'?**' – not quite, since MF forgetting forces all Q^{MF} values to relax towards a particular value. The seemingly counter-intuitive asymmetry in this effect (i.e., markedly optimistic estimates of sub-optimal actions and slightly pessimistic estimates of optimal actions) is due to the behaviour of the model – that is, the actions that it chooses and the average reward it experiences – and not in the exact mathematical formalism of our algorithm. Therefore, we hope you will agree that the term 'forgetting' is appropriate.*

> **Regardless, this was a very impressive modeling paper, and I am grateful to have had the opportunity to review it. I hope my comments were constructive.**

We hope that the above explanations were clear and addressed all your questions. Thank you for kind words and helpful comments.

Reviewer #2

> **In this manuscript, Antonov and colleagues investigates the effect of forgetting in a DYNA agent that selects optimal experiences for replay. Through various simulations and model fits to subject behavioral data, the authors explore two types of forgetting: the forgetting of action values, and the forgetting of action outcomes. They find that, under a set of modeling assumptions, a forgetful DYNA agent will tend to prioritize the replay of suboptimal actions and not of optimal actions. They then re-interpret the findings of a previous MEG study to suggest that subjects, who exhibited substantial forgetting, might have been replaying optimally when replaying suboptimal actions.**
>
> **The topic addressed is timely, given the large number of recent studies investigating the content and function of replay in rodents and humans. In particular, the elegant modeling exercise presented in the article might offer new light into existing replay data that, at first blush, may have appeared inconsistent with a normative view. However, while the authors clearly conducted a careful analysis of how forgetting might affect optimal replay, the presentation of their findings in the paper was rather confusing. Accordingly, below I present a few questions, comments, and suggestions that might improve the clarity of the paper to a broad audience.**

Thank you for your kind assessment. We apologise for the lack of clarity. We have edited the paper substantially in the light of your comments.

> **MAJOR COMMENTS:**

1. First and foremost, the paper uses many jargons and abbreviations. Please consider simplifying the language and reducing the number of abbreviations.

We are sorry for the inconvenience caused by our use of abbreviations. We now confine ourselves to just six in the main text sections: reinforcement learning (RL), model-free (MF), model-based (MB), model-influenced (MI), magnetoencephalography (MEG), and individual flexibility (IF). Additionally, we added a glossary to help readers familiarize themselves with the terms we are using. We also attempted to edit the paper to make it as clear as possible.

2. The paper presents an enormous amount of information from the very beginning of the Results section. I suspect that any reader not familiar with both Eldar et al (2020) and Mattar & Daw (2018) would have difficulty understanding the model. This might be mitigated with a clearer and more organized structure. One possibility is to organize the simulations in increasing levels of complexity, such as the following: (i) no replay; (ii) no replay with forgetting of values; (iii) normative replay with no forgetting; (iv) normative replay with forgetting of values; (v) normative replay with forgetting of the transition function (i.e. where the discussion action entropy would fall); (vi) normative replay with both forgetting of value and of the transition function. A structure such as this might help the reader understand how each factor, individually, contributes a different pattern reflected in behavior. Other structures are also possible, but the general aim should be to introduce the model components slowly such that any naive reader is able to understand it.

Thank you for pointing out the potential source of confusion; we are sorry that our model description was insufficiently clear. It is an excellent idea to provide the readers with a clear demonstration of each individual model component and their respective contributions to the resulting behaviour.

Having considered several formats according to your suggestion, we found that adding an extra paragraph (lines 203-216), along with an associated figure (Figure 3 on page 7), worked out best. We attach the newly added figure below:

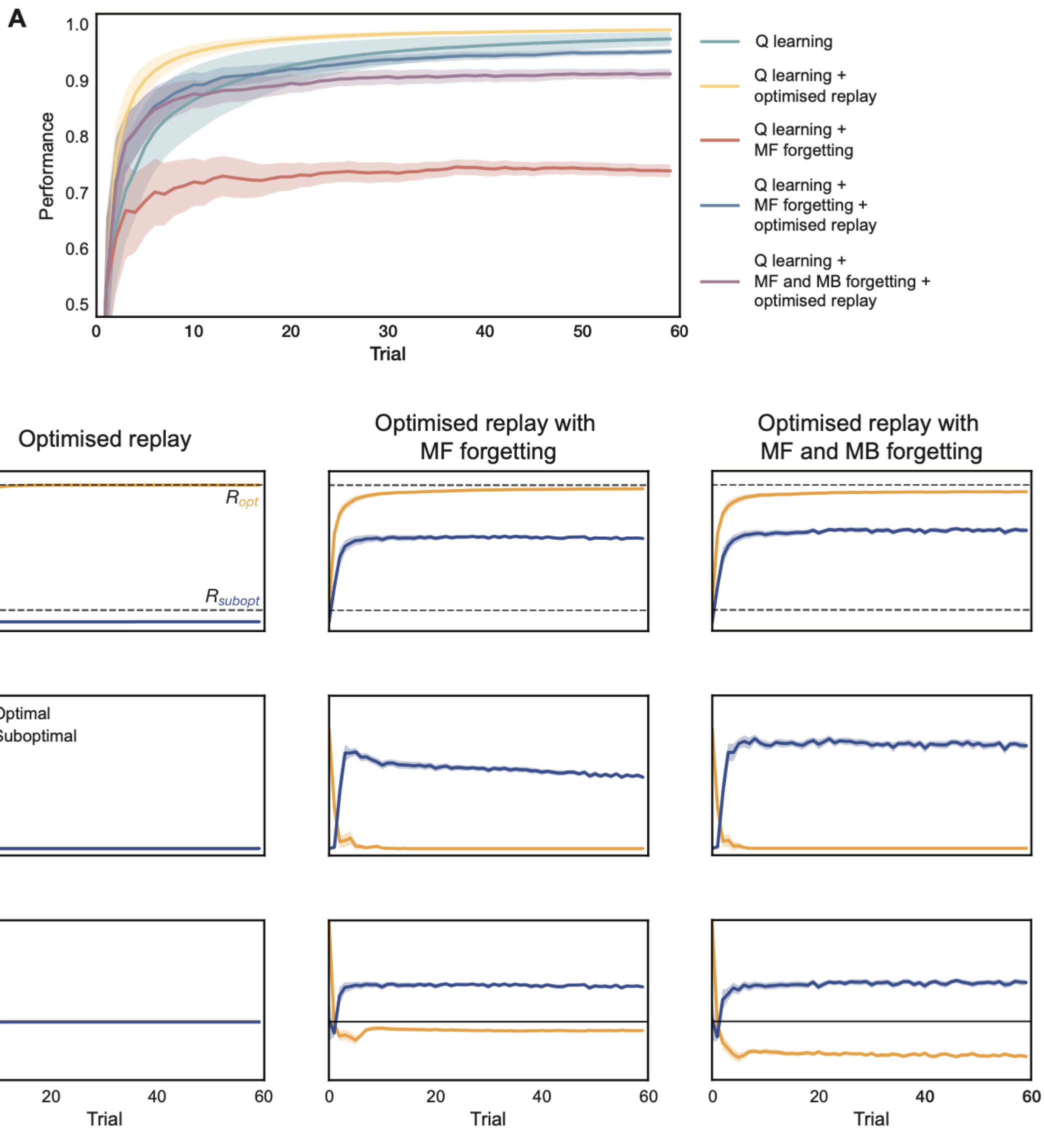

First, the figure demonstrates that agents with optimal replay significantly outperform their non-replaying counterparts (see panel A). Second, panel B highlights that MF forgetting implies a situation in which continual learning is required to maintain sound performance: without MF forgetting (leftmost plot), Q^{MF} values quickly converge such that the agent can act optimally without additional learning; MF forgetting (middle plot), however, necessitates additional off-line learning (for instance, by means of optimal replay) for the agent to maintain optimal behaviour. Finally, additional MB forgetting limits the extent to which the agent can benefit from optimal replay to help ameliorate MF forgetting (this is shown as a comparison between performance curves in panel A). Panels C and D provide additional information about the amount of replay in which the agents decide to engage and how this decision is affected by gain estimates, respectively.

We hope that such a structure helps explain the contribution of each individual model component at a suitable level of descriptive detail for its position relatively early on in the paper.

3. Forgetting is modeled as (i) MF values decaying towards a global average; and (ii) Transitions becoming more uniform. My understanding was that, in the absense of forgetting, the proposed model would reduce to the optimal model. Is that correct? If

so, it would beneficial to state that explicitly in the paper. If not, it would be important to explain why.

That is indeed correct (to clarify: Q^{MF} values relax towards the global experienced average reward). We made the relation to the optimal model more explicit in the newly added paragraph (lines 223-224):

'such an unforgetful agent precisely implements optimized replay as suggested by Mattar and Daw 2018 [citation]'

4. What would be the effect of considering also the forgetting of the (stimulus-specific) rewards?

This is an interesting question. We should note that subjects did undergo extensive training on image-reward associations before starting the main task and, moreover, the subjects were quizzed at the end of the task, which 'validated that subjects had explicit recall for both sets of image-reward associations...' (Eldar et al. 2020).

However, purely from a modelling perspective, the pattern of replay would depend on the nature of the forgetting. If, for instance, the stimulus-specific rewards were forgotten towards the average experienced reward (assuming no MF forgetting), then the agent would not engage in detectable replay. Since the agent doesn't choose sub-optimal actions often enough (and hence has little opportunity to learn about those on-line towards the forgotten stimulus-specific rewards), its Q^{MF} values for those actions would be lower than the Q^{MB} values – and hence the estimated gain would be negative; Q^{MF} values for optimal actions would similarly be, in general, lower than their corresponding Q^{MB} values (with MB forgetting), and thus the gain would also be estimated to be negative. By contrast, pessimistic replay would prevail so long as the stimulus-specific rewards are forgotten towards a value which is less than the average experienced reward (with our form of MF forgetting).

We now comment on this in the discussion (lines 607-614) as follows:

'Finally, for consistency, we borrowed some of the assumptions made in the study of Eldar et al. 2020. In particular, we did not not consider other sources of uncertainty which could have arisen from, for instance, forgetting of image-reward associations (since subjects were extensively pre-trained and, furthermore, Eldar et al. 2020 validated an explicit recall of those associations in all participants upon completion of the task). Nevertheless, we note that such a mechanism could have impacted the observed pessimized pattern of replay. Pessimistic replay would prevail so long as the stimulus-specific rewards are forgotten towards a value which is less than the average experienced reward (with our form of MF forgetting).'

5. Similarly, how important is the assumption that MF values decay towards a global average? Would the simulation results be different if the decay was, instead, towards a subject-specific baseline?

MF values decay towards the global average reward experienced by each subject, which of course differed between subjects depending on the performance they achieved in the task. Hence, MF values do in fact relax towards subject-specific baselines. This is important – if they relaxed

towards a baseline that was independent of the subject's own average reward, then there could either be too much or too little replay.

We added an additional emphasis on this effect in the following paragraph (lines 285-291):

'As the agent learns the optimal policy, the average reward it experiences becomes increasingly similar to the average reward obtainable from optimal actions in the environment. As a result, there is little room for MF Q-values for optimal actions to change through forgetting; on the other hand, MF Q-values for sub-optimal actions are forgotten towards what is near to the value of optimal actions to a much more substantial degree. Thus forgetting towards the average reward the agent experiences (provided that it accumulates with learning) is optimistic in a way that favours replay of sub-optimal actions.'

> **6. I confess that I did not understand the selection of the very peculiar form for the the softmax in 2-step choices (equation [3]). What is the reasoning behind using both Q-values in 2-move trials, instead of only the Q-value for 2-move sequences (which, according to [8], should converge to the total reward obtained in 2 steps)? How different would the simulation results be if only Q^{MF2} was used? Similarly, why is Q^{MF2} used in equation [5], instead of only Q^{MF1}? How dependent are the simulation results on this assumption?**

We chose this specific policy form for 2-move choices because the exact same equation was used by Eldar et al. 2020 – and we aimed to keep our models as close as possible so as to allow for valid parameter comparison. In the original report of Eldar et al. 2020 this choice was inspired by several factors: i) the task was designed in such a way so that some optimal moves in 1-move trials were, by contrast, sub-optimal as first moves in 2-move trials; ii) individual flexibility indices calculated by Eldar et al. 2020 clearly demonstrated that (the less flexible) subjects nevertheless more frequently (and thus incorrectly) succumbed to selecting 1-move optimal choices as first moves in 2-move trials. Therefore, the motivation for using both Q values in 2-move choices was to allow for such biased interference between the 1- and 2-move learnt values.

If only Q^{MF2} values were used in selecting first moves in 2-move trials, then the model would not have been able to capture individual flexibility indices of the subjects. Q^{MF2} values appear in equation 5 according to a similar logic – so as to incorporate the (potential) bias of first moves on choosing second moves in 2-move trials (e.g. since backtracking was not allowed).

Most importantly, Eldar et al. 2020 conducted a very thorough algorithm comparison (in the 'Additional algorithms' section of the original paper, page 18), whereby the final algorithm (with the said decision rules) was found to fit the subjects' data better than other tested algorithms (as measured by the Bayesian Information Criterion), including the ones that chose moves based solely on Q^{MF1} or Q^{MF2} values.

> **7. In equation [13], the authors assume that Q^{MB} values for 2-move sequences are calculated on-policy. Why not assume optimal behavior at the 2nd action (i.e. using a max operator instead of pi in [13]), and how important is this assumption?**

We assumed the on-policy nature of 2-move Q^{MB} values in order to be able to analyse the replay of specific 2-move sequences – for instance, to be able to distinguish between the choice to replay

(tomato, left, left) from (tomato, left, down) – as Eldar et al. 2020 were clearly able to do from the MEG data.

When looking at equation 13, however, thanks to your notice, we realised that \pi() should not be in that equation – it is an unfortunate artefact of our previous model design. We are terribly sorry for this oversight. We re-ran the fitting and simulations with the correct equation and found no significant changes in our results – i.e., all the claims that we previously made still hold. We now plot the new data simulated with the new parameter fits.

The only alteration concerns one parameter that we previously described as being significant in determining the individual flexibility of model-influenced subjects – namely, the gain threshold, which did not come out significant with the new parameter fits.

The final equation 13 is therefore:

$$Q^{MB2}(s_1, m_1, m_2) = \sum_{s'} T(s_1, m_1, s')[R(s') + \sum_{s''} T(s', m_2, s'')\, R(s'')]$$

which is a correct formula for 2-move Q^{MB} values.

> **8. In line 781, the author state that the online forgetting parameters used a beta prior with alpha=6 and beta=2, which has a mean of 0.75 and a standard deviation of only 0.14. I found this choice of prior to be rather specific, contrasting sharply with the more uninformative choices for all other priors. I would have loves to see how the simulations would have turned out if a uniform prior had been used. Would subjects still have been found to be forgetful?**

First, when looking at our algorithm description, thanks to your comment, we noticed that the parameters for the beta prior were quoted the wrong way around, for which we are most apologetic. The original semantics of the quantities had been expressed in terms of remembrance instead of forgetting, and so we had the opposite form.

Second - thank you also for pointing out the issue with specifying our prior distributions for the fitting procedure. We apologise for not detailing our fitting procedure well enough, which has unfortunately led to a misinterpretation of the role played by the specific shape of these distributions. In fact, as we detail below, this prior (which we now refer to as the initialization distribution) is just for the start state of our Markov chain Monte Carlo method - the actual prior distribution used within the chain is uniform, as you suggest. The main role of the initialization distribution was to accommodate subjects who were inferred as engaging in very substantial amounts of replay.

To be specific: in fitting our model parameters, we used the Approximate Bayesian Computation (ABC) approach, which is a simulation-based procedure for approximating the joint posterior distribution over the parameters. More specifically, we used the Metropolis-Hastings ABC algorithm (algorithm 1 on page 25), which constructs a Markov chain of parameter samples $\{\theta_i\}_{i=0,\dots,N}$ from the approximate joint posterior distribution.

In each iteration, the algorithm samples θ^* *from the proposal Gaussian distribution* q *centred at the parameter sample which was accepted in the previous iteration,* $q(\theta^*) = N(\theta_{i-1}, \Sigma_i)$. *The covariance matrices* $\{\Sigma_i\}_{i=0,\dots,N}$ *were diagonal, with pre-defined and exponentially decreasing entries for each iteration.*

The acceptance criterion for each proposed parameter sample θ^* *was determined by a synthetic likelihood function* $\rho(X, Y)$, *which we took to be the root-mean-squared deviation between X, the performance data simulated with the proposed parameter sample* θ^* *, and Y, the true performance data for each human subject.*

The acceptance probability for the Metropolis-Hastings ABC algorithm is:

$$\alpha = min(1, \frac{\pi(\theta^*)\, q(\theta_{i-1} \mid \theta^*)}{\pi(\theta)\, q(\theta^* \mid \theta_{i-1})}) \textit{if } \rho(X, Y) < \varepsilon_i \textit{ and 0 otherwise.}$$

Where $\varepsilon_i \in \{\varepsilon_i\}_{i=0,\dots,N}$ *is the likelihood threshold, which, in our case, was also exponentially decreasing for each consecutive iteration; and* $\pi(\theta)$ *is the assumed prior distribution of the parameter sample. This prior is indeed uniform (and so cancels out in the acceptance step). Thus, since our proposal distribution* q *was Gaussian (and hence symmetric), the acceptance probability for our algorithm can be further simplified to:*

$$\alpha = 1 \textit{if } \rho(X, Y) < \varepsilon_i \textit{ and 0 otherwise.}$$

Therefore, in each iteration, the proposed parameter sample θ^* *was accepted only if it resulted in the likelihood* $\rho(X, Y) < \varepsilon_i$, *regardless of the originally specified initialisation distribution. Furthermore, beginning with the second fitting iteration, the proposal distribution had a relatively high variance of 0.5 which then decreased exponentially. Such high variance during the very first few iterations ensured that the proposed parameter samples covered the entire support (in the case of distributions with bounded support, such as the Beta distribution).*

The figure below shows the values that fall within 1 standard deviation of the proposal distribution mean for every parameter (which, in this example, was set to 0.5) as the fitting iterations proceed:

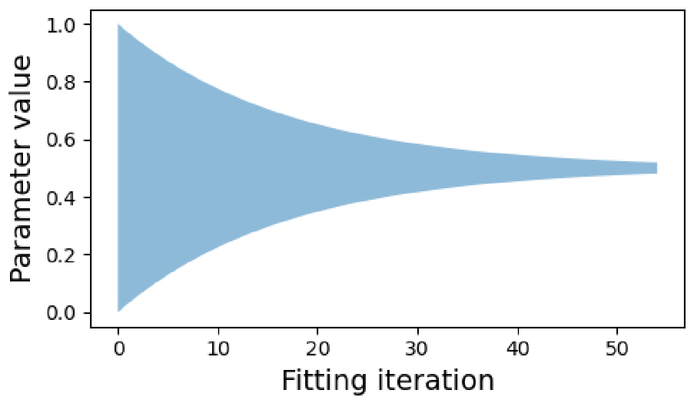

Thus, as made clear in the above figure, our fitting protocol covers the entire distribution support at the very outset of the procedure.

Finally, below we show example trajectories (in the parameter space) sampled by our procedure that led to the final parameter fits for several subjects:

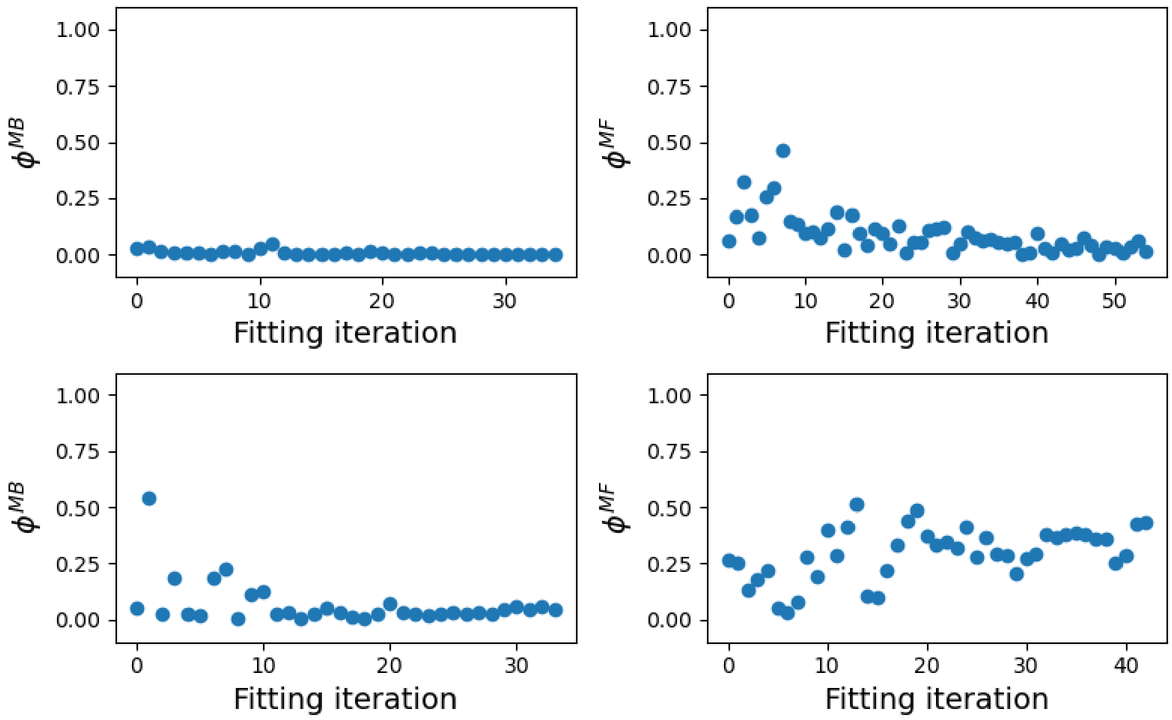

The figure clearly demonstrates that our fitting procedure did not restrict the variability in the parameter guesses in the first few iterations (as can be seen, for instance, from the bottom-right plot).

The specific initialization distribution parameters that you mentioned were chosen to improve the acceptance rate in the very first iteration of the fitting procedure (and hence reduce the computational cost of simulating many proposed samples) for subjects that achieved very high performance (since they extensively engaged in replay) – indeed, one can see that the parameter values in the top-left plot were constrained to a very narrow range of permissible values.

Therefore, to be more precise and avoid the potential ambiguities, as noted above, we renamed our 'prior distributions' to the 'initialisation distributions' – since that is what those effectively are; moreover, we now explicitly mention the uniform nature of prior distributions assumed in our fitting procedure in lines 818-827.

MINOR COMMENTS:

9. Line 192-196: "we find that at high MF forgetting, replay confers a noticeable performance advantage to the agent provided that MB forgetting is mild (as can be seen from the curvative of the contour lines in Fig 2D)". However, Fig 2D only compares replay models with different forgetting rates. Does replay really confer a

performance advantage in comparison to an agent that performs no replay? In other words, does replay with substantial forgetting hurt performance, and why/how?

Relating to your Major comment #2, we have now included an explicit comparison between agents with and without optimal replay and forgetting which shows that such replay does indeed improve performance (in agents both with and without forgetting).

The issue of how replay can hurt performance is considered in great detail in the section where we discuss subject-specific parameters implied by our modelling. More specifically, in lines 505-509 and 518-523 we describe under what circumstances replay can hurt and exemplify it with supplementary figure 5 which shows an example move from a subject which, according to our model prediction, significantly hurt him/herself by engaging in (subjectively) optimal replay:

'Moreover, we note that extensive training of MF values by a forgetful MB policy (as a result of setting the gain threshold too low) can be dangerous. For instance, our modelling predicted the example subject in S5 Fig B to be close to complete ignorance about the transition structure of the task (S5 Fig B), yet the subject, as our agent predicted, still chose to engage in replay which significantly hurt his performance.'

We decided to refrain from elaborating on this possibility when introducing the model (although the distinction between subjective and objective value estimates is made very explicit throughout) – since this would have made the simple example simulations we present in the beginning much more difficult to grasp.

10. Fig 3A is extremely difficult to parse. Same for Fig 3B (e.g. what do the axes mean?).

We are sorry that Fig 3 was hard to interpret.

Fig 3A shows how Gain estimate is affected by the difference between the agent's current Q^{MF} values and the model-estimated Q^{MB} values proposed for replay. Fig 3B shows how MF forgetting affects the steady-state Q^{MF} values after a considerable amount of learning (the different bar shadings indicate the different severity of MF forgetting).

We changed the axes labels appropriately so that, hopefully, it is now clearer. The modified figure is attached below:

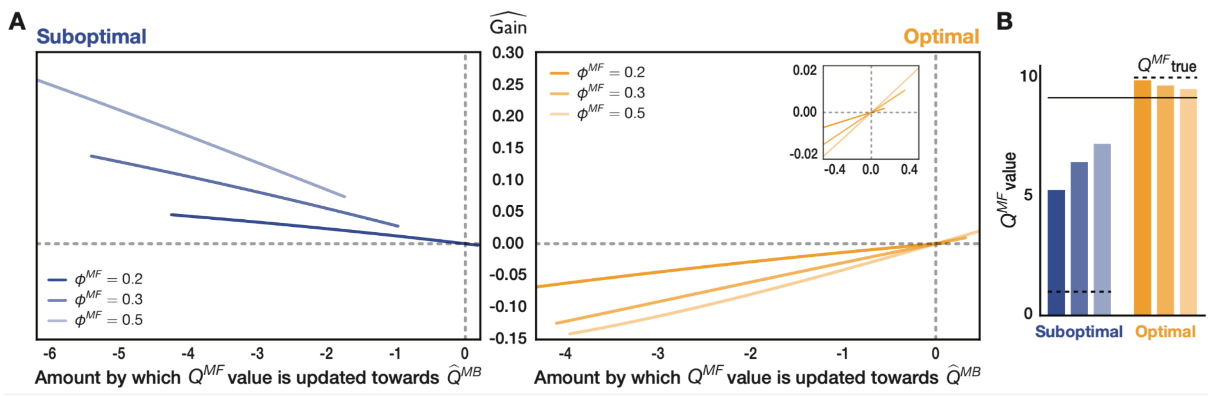

11. Avoid using asterisks in actions (as in equation [4]), which in RL is generally used to indicate an optimal action.

Sorry – we adhered to the notation assumed by Eldar et al. 2020. It is indeed misleading and thus we changed it accordingly.

12. Similarly, in equation [4] and others, it was not clear why Q^{MF2} sometimes had three arguments and sometimes two.

The two-argument Q^{MF2} *refers to a marginal value of first moves (that is, when choosing a first move in 2-move trials the agent marginalises the learnt 2-move value function over the possible second moves according to its current MF policy). We made this clarification explicit in the text in lines 672-674:*

'The two-argument Q^{MF2} *value used when choosing a first move therefore corresponds to a two-move MF Q value where the second move options are marginalised out with respect to the agent's policy.'*

13. R(s) is used to indicate a reward model in equations [12-13], and a sampled reward in [7-8]. Consider using different notation in each case.

Similarly to Eldar et al. 2020, we assumed that the subjects had learnt image-reward associations well enough (which was assessed after extensive training in the original report) and thus R(s) refers to the true reward model in all cases throughout the manuscript.

14. On line 782, the authors state: "All the other parameters had gamma priors with location 1 and scale 1". Please list all such parameters explicitly.

We have done this.
