# Supplementary material for: Optimism and pessimism in optimised replay
Source: PLoS Comput Biol. 2022 Jan 12;18(1):e1009634. doi: 10.1371/journal.pcbi.1009634 (PMC8809607; doi:10.1371/journal.pcbi.1009634)
Supplement: Attachment — Submitted filename: Responses2.pdf [file pcbi.1009634.s011.pdf]

Reviewer #1

I thank the authors for addressing my comments and for a comprehensive revision.

Thank you very much for your detailed feedback which has helped us significantly improve the paper.

I believe most of the confusion was due to the prominent use of 'forgetting' in a context I was unfamiliar with. I would ask the authors to

(1) cite other work that that uses this definition of forgetting (i.e. decay to average reward rate) since they claim it is the standard and ...

We apologise for any misunderstanding.

The mathematical formalism of forgetting that we employed in our work is quite common; for instance, Barraclough et al. (2004) introduced a modified forgetful Q-learning algorithm to account for the observed influence of reward history on monkeys' choices in a matching pennies game. Later, Ito & Doya (2009) showed that forgetful Q-learning agents fit rats' decision strategies better than the standard unforgetful Q-learning algorithm. In a more recent study, Toyama et al. (2019) also showed that forgetful Q-learning algorithms were favoured in a model comparison procedure with the free parameters fit to the human subject data (in a version that allowed for the forgetting of values towards an arbitrary threshold). Decay to the average reward rate is perhaps less common - but this reward rate, which is also considered in terms of the opportunity cost of time, is justifiable as a form of invariant for planning (unlike an arbitrary number such as 0).

We agree that it would be helpful to cite those works mentioned above, in which the authors also investigated the role of forgetting. This is now mentioned in the discussion in lines 567-572:

'We studied these phenomena in the task of Eldar et al. (2020), where it was initially identified. Forgetting had already been identified by Eldar et al. (2020) (in fact, a number of previous studies have also noted the beneficial effect of value forgetting for fitting behavioural data [Barraclough et al. (2004), Ito and Doya (2009), Toyama et al. (2019)]); however, it played a more significant role in our model, because we closed the circle of having MB replay affect MF values and thus behaviour. By not doing this, forgetting may have been artificially downplayed in the original report (S2 Fig A, B).'

And we also now refer to the average reward rate employed in our modelling in the response to your next comment.

(2) comment on whether this is a proposal for what forgetting is defined as in a psychological context, or if this is specific jargon used in technical reinforcement learning settings that does not necessarily bear on (folk) psychological notions of forgetting

Our notion of forgetting is indeed inspired by the reinforcement learning terminology, since forgetting in our case operates on the learnt expected reward values. Furthermore, as noted above, it has appeared in the computational and behavioural neuroscience literature, used for modelling choices and neural responses in RL tasks. In terms of the psychology of forgetting, the task that we are modelling is unfortunately not very useful for dissociating decay, or prospective or retrospective interference, since trial start- and end-points were not chosen with this in mind. We also lacked the means to test longer-term consolidation. We have now added this caveat to the text (lines 573-579).

'Additionally, we note that our forgetting mechanism first arose in the literature on computational and behavioural neuroscience; average reward rate, for instance, has been found to be an important facet of behaviour -- being used as the opportunity cost of time in accounts of controlled vigour [Niv et al. (2007)]. Unfortunately, the task is not very well adapted to address broader psychological questions of forgetting [Wixted (2004)], since opportunities for decay and prospective and retrospective interference all abound in the trial-type sequences employed. Furthermore, longer-term consolidation was not a focus.'

Reviewer #2

> **The structure and clarity of the paper has greatly improved. Thank you very much for taking the time to implement these changes. I am sure that this will increase the impact of the paper.**

We are very happy to hear that. Thank you for the kind words and the effort to help us improve the paper.

> **I have only a few final minor suggestions left:**

> **Line 111: The caption says "Unfilled hexagons show epochs which contained trials without feedback", but the figure shows no unfilled hexagons.**

We apologise for this oversight – this has been changed.

> **Fig 3B: Please add the legend indicating the meaning of the yellow and blue lines (currently, there's only a label for the dashed lines)**

The legend for the yellow and blue lines originally appeared in a different panel; we have changed this according to your suggestion (the lines are now also defined in the figure caption).

> **Line 237: In the previous sentence (line 235-236), the authors say "provided that MB forgetting is mild", followed by "This means that despite moderate uncertainty in the transition structure, the agent is still able to improve its MF policy and increase the obtained reward rate." Don't the authors mean: "as long as there is little uncertainty in the transition (...)"?**

Exactly, we just had the emphasis the other way around. Thank you for pointing us at this sentence. We changed it to (lines 239-241):

‘We find that at high MF forgetting, replay confers a noticeable performance advantage to the agent provided that MB forgetting is mild (as can be seen from the curvature of the contour lines in Fig 2D). This means that, by engaging in replay, the agent is able to (on average) improve its MF policy and increase the obtained reward rate as long as there is little uncertainty about the transition structure.’
